# Ghrelin Levels and Postnatal Growth in Healthy Infants 0-3 Months of Age

**DOI:** 10.4274/jcrpe.v2i1.34

**Published:** 2010-12-08

**Authors:** Kürşad Fidancı, Cihan Meral, Selami Süleymanoğlu, Özgür Pirgon, Ferhan Karademir, Seçil Aydınöz, Halit Özkaya, Mustafa Gültepe, İsmail Göçmen

**Affiliations:** 1 Department of Pediatrics, GATA Medical Faculty, İstanbul, Turkey; 2 Department of Pediatric Endocrinology, GATA Medical Faculty, İstanbul, Turkey; 3 Department of Biochemistry, GATA Medical Faculty, İstanbul, Turkey; +90 332 323 67 09/5300ozpirgon@hotmail.comKonya Research and Training Hospital, Department of Pediatric Endocrinology Meram, Konya, Turkey

**Keywords:** growth, ghrelin, weight gain, small for gestational age, large for gestational age

## Abstract

**Objective**: The effect of ghrelin on growth of the newborn has long been argued, but not fully clarified. In this study, we aimed to investigate the relationship between ghrelin levels and growth parameters in the first 3 months of life.

**Methods**: The study included 60 babies (27 girls and 33 boys) born at gestational ages between 38-42 weeks. The newborns were divided into three groups according to the Lubchenco curves as: small for gestational age (SGA), appropriate for gestational age (AGA) and large for gestational age (LGA). The relationship between ghrelin levels and growth parameters in the third month was investigated.

**Results**: Ghrelin concentrations were significantly higher in SGA (2.4±2.6 ng/dL) babies than in AGA (1.3±0.9 ng/dL) and LGA (1.0±0.8 ng/dL) babies. The lowest ghrelin levels were in the LGA group. In SGA infants, ghrelin concentrations were inversely correlated with change in weight (r=-0.577; p=0.001), change in length (r=-0.361; p=0.005), and change in head circumference (r=-0.387; p=0.002).

**Conclusion**: The results show that at age 3 months, SGA infants had higher ghrelin levels than AGA and LGA infants. Our findings indicate that ghrelin may be involved in the process of catch-up growth in these infants.

**Conflict of interest:**None declared.

## INTRODUCTION

Ghrelin is a peptide hormone, which has an effect on growth hormone secretion and energy balance. It also has a central effect on eating behavior and regulation of body weight. When first discovered, it has been considered as a growth hormone secretagogue. In recent publications, attention has been drawn to its effects on appetite and weight regulation (1). More recently, ghrelin has been detected in cord blood (2). 

Ghrelin may be implicated in the stimulation of appetite and growth hormone release in both rodents and humans (3, 4). Human subjects, who were given ghrelin by iv injection, reported increased ratings of hunger on a visual analog scale (4).

Recent studies have reported the potential role of ghrelin in adaptation to intrauterine malnutrition (5). Lower ghrelin levels have been related to slower growth in small for gestational age (SGA) infants, and infants with lower cord ghrelin levels have been reported to gain weight more slowly from birth to 2 years (6). 

The aim of this study was to investigate the relationship between ghrelin and growth parameters at birth and at age three months in healthy infants.

## METHODS

Newborn infants born in GATA Medical Faculty Hospital between July 2005 and March 2006 by normal spontaneous vaginal delivery were enrolled in the study. Gestational age was assessed by maternal last menstrual date and confirmed by Dubowitz scoring (7). We excluded infants whose mothers had any clinical conditions such as diabetes mellitus or, parathyroid disease, skeletal, renal and gastrointestinal dis-orders. None of the participating mothers smoked during pregnancy. Women who gave birth to singleton term babies were recruited in the delivery room. A total of 60 babies (27 girls and 33 boys) were included in the study. The infants were divided into the following three groups using the Lubchenco intrauterine growth curves (8):

**Small for Gestational Age (SGA)**: This group included 20 babies with birth weights <10^th^ percentile. 

**Appropriate for Gestational Age (AGA)**: This group included 20 babies with birth weights between 10^th^ and 90^th^ percentiles.

**Large for Gestational Age (LGA)**: This group included 20 babies with birth weights >90^th^ percentile.

All newborns included in the study were healthy infants and products of consecutive deliveries. Their mothers had had no remarkable complications during pregnancy. None of the infants had congenital malformations, chromosomal abnormalities, or intrauterine infections. Their 5^th^ minute Apgar scores were ≥8 and their physical examinations were normal. Anthropometric measurements (length, weight, head circumference) were made on each infant and blood samples were taken. 

Birth weight and birth length were obtained from each neonate immediately after birth. The infants were weighted naked on an electronic scale. For length measurements a measuring board (head portion stable; feet portion mobile) was used. Measurement of head circumference was done on day 2 to allow for resolution of edema and head molding. Head circumference measurements were made by passing a non-stretch measuring tape from the glabella through the occipital protuberance. Approval for the study was obtained from the GATA Medical Faculty Ethics Committee on December 1, 2005 (48^th^ session). Informed consent was obtained from each mother before the blood samples were taken from their infant. 

All infants were exclusively breast-fed throughout the study. 

The blood samples (venous blood samples, 3-5 mL) from each infant were taken in the early neonatal period (first 7 days) and in the 3^rd^ month, after a period of fasting (approximately 90 minutes after feeding), between 9.00 am and 9.30 am. All samples were assayed together at the end of the study, so measurements of growth were recorded blind to the hormone levels. The samples were collected in ethylenediaminetetraacetic acid (EDTA) tubes. Within 1 hour, the chilled samples were centrifuged, the plasma was removed and stored at -70°C until being assayed. Ghrelin levels were measured with Ghrelin Human EIA kit (S-1222; Peninsula Laboratories Inc., Member of the Bachem Group, California, USA) by using ELISA (Enzyme-linked immunosorbent assay) method. A Microplate Reader RT-2100 C device was used in the readings.

**Statistical Analysis**

The data were expressed as means±SDs. The Kolmogorov-Smirnov test was applied separately for the groups to check the normality of the variables. Differences in the means of variables were tested using both parametric and non-parametric tests depending on the distribution of the variables between the groups. The changes in anthropometric measurements (length, weight and head circumference) and serum ghrelin levels between baseline values and values obtained at 3 months were separately tested for the three groups using the paired-sample t-test. Pearson’s correlations were used to examine relationships among clinical growth-related parameters and hormone levels. Statistical significance was taken as p<0.05. All statistical analyses were performed using the Statistical Package for Social Sciences (SPSS/Windows version 11.0, SPSS inc., Chicago, IL, USA).

## RESULTS

Table 1 shows the findings of the infants at birth, at 3 months of age and the changes in these variables over time. There were no differences between males and females in terms of anthropometric data in any of the 3 groups. There were also no significant gender differences in ghrelin concentrations in the neonatal period or at 3 months of age. 

**Figure 1:**
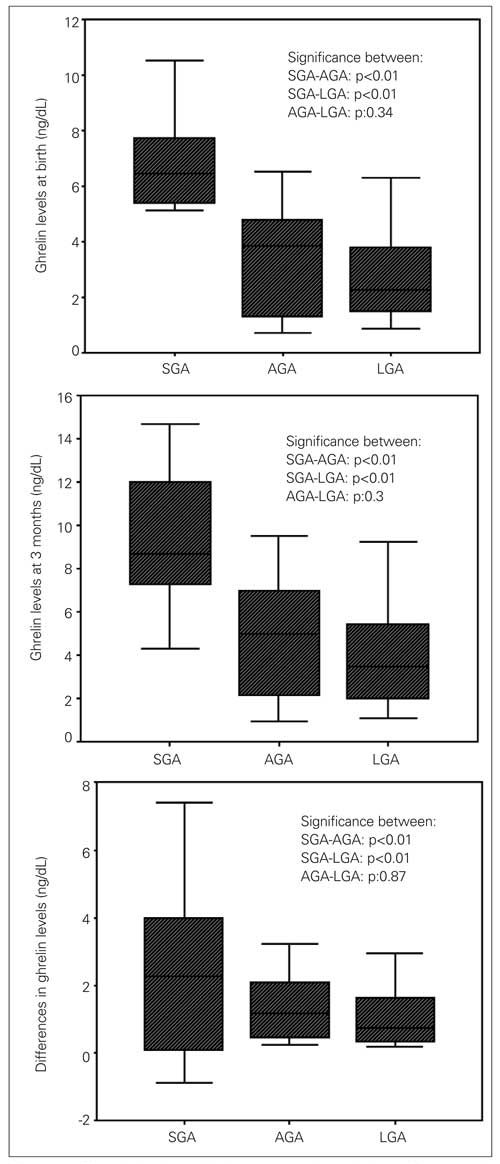
Ghrelin levels at birth, at 3 months and the differences in ghrelin levels in the three groups SGA: small for gestational age, AGA: appropriate for gestational age LGA: large for gestational age

While there were no significant differences between their gestational ages, as expected, significant differences existed between the groups at baseline in their anthropometric indices (Table 1). Ghrelin was significantly higher in SGA and lower in LGA infants compared to AGA infants, as depicted in Table 1 and also in Figure 1. 

At age 3 months, weight gain was significantly higher in the AGA group babies than in the other groups (2837±631 grams in SGA, 3186±680 grams in AGA and 2726±637 grams in LGA group; p<0.001 between AGA and LGA groups). However, the percentage of weight gain was significantly higher in SGA babies than in LGA babies (124% vs. 64%). 

The change in ghrelin levels was significantly higher in SGA (2.4±2.6 ng/dL) babies than in AGA (1.3±0.9 ng/dL) and LGA (1.0±0.8 ng/dL) babies. The least change in ghrelin levels was in the LGA group. Ghrelin concentrations were inversely correlated with change in weight (r=-0.577; p=0.001), change in length (r=-0.361; p=0.005) and change in head circumferences (r=-0.387; p=0.002) in the SGA group, but not in the other groups.

Ghrelin levels and changes are also depicted in Figure 1.

**Table 1:**
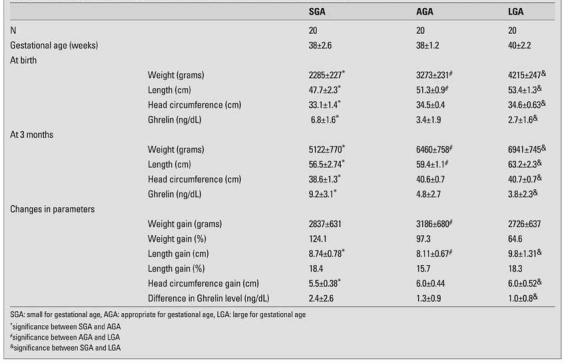
Anthropometric data and ghrelin levels at birth and after 3 months in the three groups

## DISCUSSION

Ghrelin has been proposed to be a hormone involved in pre- and postnatal growth, and its serum levels have been considered of prognostic value for postnatal catch-up growth. In some studies ghrelin levels were reported to be associated with anthropometric parameters (9, 10). Also in our study, ghrelin levels of SGA infants were found to be higher when compared to AGA and LGA infants in the early neonatal period and in the third month of life. These findings indicate that ghrelin takes part in the energy balance and is affected from intrauterine nutritional environment. In addition, we found that higher ghrelin levels are associated with more weight gain over the first 12 weeks of life in SGA infants. This finding implies that SGA infants who are growing faster have a greater increase in ghrelin levels from fasting values compared with AGA and LGA infants growing at a slower pace.

High ghrelin concentrations sustained by a continuous infusion have been associated with increased weight and adiposity in mice (11). However, other researchers did not report an effect of daily injections of ghrelin on weight gain during the first postnatal week in neonatal rats, and the ghrelin-null mouse model fails to support a role for ghrelin in postnatal growth (12, 13). The way ghrelin might influence weight gain in early infancy in humans is unclear, but a simple explanation would be that lower ghrelin concentrations are associated with reduced appetite, resulting in lower nutritional intake. High circulating ghrelin concentrations have been described in SGA neonates, and SGA infants showing slower weight gain over the first year of life had lower ghrelin levels after an iv glucose load (3, 14). These findings suggest that ghrelin may be an important regulator of weight gain in early infancy.

Ghrelin levels might be increased to induce appetite and to form a positive energy balance after birth-at a time when energy supply from the placenta ceases and breast-feeding starts. Yokata et al (15) and Farquhar et al (5) found significantly higher ghrelin levels in term SGA infants compared to AGA and LGA infants in the postnatal period. Higher ghrelin levels in the cord blood of SGA infants compared to AGA infants, and higher postnatal ghrelin concentrations compared to cord blood values in term newborns were reported (1, 16). Savino et al (17) found a positive correlation between the infant’s age and ghrelin concentration. We have detected a positive correlation between ghrelin concentrations and weight gain in the postnatal period in term infants. Our findings are compatible with the literature and suggest that ghrelin might play a role in growth in this period of life. 

There are reports which indicate that ghrelin levels may be affected by acute changes as well as long-term changes in energy balance. Plasma ghrelin levels vary as a consequence of the adaptive response to these circumstances. Bellone et al (18) demonstrated that, unlike adults, ghrelin levels did not tend to decrease postprandially in children, and concluded that the ghrelin system functions somewhat differently in childhood. Savino et al (17) found that in infants, ghrelin levels were lower in the 0-4 month period. They did not observe any difference between breast-fed and formula-fed infants in the 4-8 month and 8-12 month periods. In our study, all infants were breast-fed. As also reported by others (17, 19), we found no correlation between gender and ghrelin levels at birth and at 3 months of age.

In conclusion, our findings indicate that in SGA babies, higher ghrelin levels at birth are associated with faster weight gain in the first 3 months of life.
